# Biocompatible PLGA-Mesoporous Silicon Microspheres for the Controlled Release of BMP-2 for Bone Augmentation

**DOI:** 10.3390/pharmaceutics12020118

**Published:** 2020-02-01

**Authors:** Silvia Minardi, Joseph S. Fernandez-Moure, Dongmei Fan, Matthew B. Murphy, Iman K. Yazdi, Xuewu Liu, Bradley K. Weiner, Ennio Tasciotti

**Affiliations:** 1 Center for Biomimetic Medicine, Houston Methodist Research Institute, Houston, TX 77030, USA; silvia.minardi@northwestern.edu (S.M.); dongmeifan@gmail.com (D.F.); matthewmurphy21@gmail.com (M.B.M.); ikyazdi@houstonmethodist.org (I.K.Y.); etasciotti@houstonmethodist.org (E.T.); 2 Division of Trauma, Acute and Surgical Critical Care, Department of Surgery, Duke University, Durham, NC 27517, USA; joseph.fernandezmoure@duke.edu; 3 Department of Nanomedicine, Houston Methodist Research Institute, Houston, TX 77030, USA; xliu@houstonmethodist.org; 4 Department of Orthopedic Surgery, Houston Methodist Hospital, Houston, TX 77030, USA

**Keywords:** BMP-2, silicon, microsphere, PLGA, bone regeneration, controlled release

## Abstract

Bone morphogenetic protein-2 (BMP-2) has been demonstrated to be one of the most vital osteogenic factors for bone augmentation. However, its uncontrolled administration has been associated with catastrophic side effects, which compromised its clinical use. To overcome these limitations, we aimed at developing a safer controlled and sustained release of BMP-2, utilizing poly(lactic-*co*-glycolic acid)-multistage vector composite microspheres (PLGA-MSV). The loading and release of BMP-2 from PLGA-MSV and its osteogenic potential in vitro and in vivo was evaluated. BMP-2 in vitro release kinetics was assessed by ELISA assay. It was found that PLGA-MSV achieved a longer and sustained release of BMP-2. Cell cytotoxicity and differentiation were evaluated in vitro by MTT and alkaline phosphatase (ALP) activity assays, respectively, with rat mesenchymal stem cells. The MTT results confirmed that PLGA-MSVs were not toxic to cells. ALP test demonstrated that the bioactivity of BMP-2 released from the PLGA-MSV was preserved, as it allowed for the osteogenic differentiation of rat mesenchymal stem cells, in vitro. The biocompatible, biodegradable, and osteogenic PLGA-MSVs system could be an ideal candidate for the safe use of BMP-2 in orthopedic tissue engineering applications.

## 1. Introduction

Autologous bone grafting remains the gold standard for spinal fusion, traumatic non-union and total hip arthroplasty complicated by osteolysis [1,2,3], yet it comes with morbidity (separate incision, graft site pain, potential infection, etc.) and might provide insufficient volumes of bone for complex or multilevel reconstruction [4]. Bone morphogenetic protein-2 (BMP-2) is a transforming growth factor known to play a key role in the development and repair of bone and cartilage [5]. Initially, it appeared to provide an ideal solution [6,7,8] to enhance bone growth but as its clinical use has expanded, multiple complications associated with BMP-2 use have come to light including local wound problems, chemical radiculitis, bony overgrowth into the canal or foramen, osteoclast activation with associated bony resorption and device displacement, and, possibly, cancer when used at very high doses in an off-label manner [1,9,10,11]. It is thought these complications were associated with the uncontrolled release and systemic distribution of supraphysiologic doses of this potent growth factor [1,12]. One promising way to avoid these complications is via the controlled local delivery of very small but effective doses using the combination of growth factors with controlled drug delivery vehicles [13,14,15,16,17].

To date, however, systems designed to provide this controlled release have been limited by: (a) a burst release phenomena which leads to similar supraphysiologic dosing, inefficiently sustained dosing, and uncontrolled delivery as that seen with delivery systems currently used clinically; (b) the inability to preserve the quaternary structure of drugs following release from the delivery system; and (c) delivery system (polymer) degradation byproducts that have a secondary negative impact on the structure of the drugs released [16,18]. Therefore, a new carrier system capable of sustained, regulated, local release of small but effective doses that do not impact BMP-2 functionality are needed to allow the avoidance of the biological complications associated with burst supraphysiologic dosing [16].

With the development of nanomaterials, several types of particles, both nano and micro, have been used as growth factor delivery carriers [19]. Of these, nanoporous silicon has emerged as a material uniquely capable of the preservation of protein stability and function with predictable degradation properties in physiologic fluids and systems [16,17,19,20,21,22]. The breakdown product, orthosilic acid, has also been shown to stimulate mineralization by osteogenic cells while retaining the ability to buffer the breakdown products of coating polymer (poly(lactic-*co*-glycolic acid), PLGA) [19,23].

Recently, by integrating the drug preserving and encapsulating capabilities of nanoporous silicon with the further controlled release capabilities achieved by polymer encapsulation (using PLGA), we optimized a double controlled delivery system for the controlled and sustained temporospatial release of growth factors [17]. The platforms consisted of the mesoporous silicon-based multistage vector (MSV), encapsulated within a PLGA microparticle (PLGA-MSV). In our previous study, we demonstrated that PLGA-MSV is able to efficiently load a growth factor (i.e., PDGF-BB) and release it in a controlled fashion in vivo, with a significant reduction of the initial burst release, while preserving its functionality (i.e., inducing vascularization) [17].

The aim of the current study was to optimize the release of BMP-2 through the PLGA-MSV delivery system and assess its effectiveness in inducing osteogenesis in vitro.

## 2. Materials and Methods

### 2.1. Preparation of PLGA-MSV Microspheres

The PLGA-MSV microspheres were fabricated by a modified S/O/W emulsion method as in our previously published studies [7]. Briefly, PLGA (Sigma Aldrich, St. Louis, MO, USA was dissolved in dichloromethane (DCM; Fisher Scientific, Loughborough, UK) to form PLGA/DCM organic phase solution (10% and 20% *w*/*v*). BMP-2 loaded particles (8 × 10^7^) were suspended into 1 mL of PLGA/DCM solutions (10% and 20% *w*/*v* respectively) by vortex mixing and sonication for 2 min. The organic phase containing the MSV particles was transferred into 3 mL of PVA (2.5% *w*/*v*) solution and emulsified for 1 min by vortex mixing. The primary emulsion was then gradually dispersed into 50 mL of PVA solution (0.5% *w*/*v*). The resulting suspension was stirred continually for 2 h under a biochemical hood, and the DCM evaporated rapidly during the stirring process. PLGA-MSV microspheres were washed with deionized water 3 times and lyophilized overnight. The freeze-dried BMP-2 loaded PLGA-MSV microspheres were then stored at −80 °C.

### 2.2. Characterization of PLGA-MSV Microspheres

The morphology of the microspheres was characterized by scanning electron microscope (SEM; Nova NanoSEM 230, FEI, Lincoln, NE, USA) and confocal microscope (Nikon A1 laser confocal microscope). The samples were sputter coated with 8 nm of platinum (Pt; Cressington sputter coater 208 HR System, Ted Pella, Inc., Watford, UK) and examined by SEM under a voltage of 3 kV, spot size 3.0, and a working distance of 5 mm.

### 2.3. Loading of BMP-2 into Nanoporous Silicon Particles (MSV)

Two hundred microliters of BMP-2 growth factor solution (Peprotech) was added into 8 × 10^7^ oxidized nanoporous silicon particles in an Eppendorf tube. The suspension was mixed throughout by vortex mixing and sonication. The tube was gently rotated on a rotator at room temperature for 2 h to allow the adsorption of BMP-2 into the MSV particles. The BMP-2 loaded particles were then spun down by centrifugation (Sorvall Legend X1R Centrifuge, Thermo Scientific, Waltham, MA, USA) (4500 rpm for 5 min), lyophilized overnight, and stored at −80 °C for future use. The concentrations of the BMP-2 loading solution and the supernatant were measured by Elisa assay to determine the amount of BMP-2 loaded into the MSV particles.

### 2.4. Evaluation of Growth Factor (BMP-2) In Vitro Release

The BMP-2 loaded PLGA-MSV microspheres (10% and 20% *w*/*v*) containing 8 × 10^7^ of MSV particles were dispersed into 0.5 mL of 1% BSA solution at 37 °C. The BMP-2 loaded PLGA microspheres (10% and 20% *w*/*v*) were used as a control. At predetermined time intervals, the suspension was spun down at 4500 rpm for 5 min and 0.5 mL of each supernatant was collected, and replaced with 0.5 mL of fresh 1% BSA solution. The amount of BMP-2 released from BMP-2 loaded PLGA-MSV microspheres was detected using an enzyme-linked immunosorbent assay kit (BMP-2 ELISA, R&D Systems, Minneapolis, MN, USA).

### 2.5. Cell Isolation and Culture

The study protocol and all operations were reviewed and approved by the Houston Methodist Research Institute’s Institutional Animal Care and Use Committee (IACUC, protocol IS00003525, 18 August 2010). All investigators complied with the National Research Council’s Guide for the Care and Use of Laboratory Animals. Male Sprague Dawley rats (*N* = 10) with an average weight of 310 g were used in the study. The rodents underwent a mandatory 48 h acclimation time prior to any surgical procedures and were housed in pairs with ad libitum water and chow until the study period began. Bone marrow stromal cells (BM-MSCs) were isolated from male Sprague Dawley rats as previously described. Briefly, femora and tibiae bones were removed from male Sprague Dawley rats (100–125 g) sacrificed by CO_2_ overdose under isoflurane anesthesia. Bones were cleaned of connective tissues, ligaments, and muscle by scalpel, washed thoroughly in phosphate-buffered saline (PBS, Invitrogen, Carlsbad, CA) containing 2% penicillin and streptomycin (P/S, Invitrogen). The proximal and distal ends of each bone were removed and the marrow was gently flushed out with PBS containing 1% fetal bovine serum (FBS, HyClone, ThermoFisher 124 Scientific, Logan, UT, USA) and 1% P/S. The diaphysis regions were crushed using a mortar 125 and pestle while submerged in PBS washes until the PBS appeared clear, indicating complete removal of the remaining marrow and perivascular cells. The total BM fraction was kept on ice for up to 1 h prior to further purification. The total BM cell fractions was counted by hemocytometer and then purified to mononuclear cells by centrifugation on Ficoll (150× *g* for 30 min without brake). The mononuclear BM populations were counted, resuspended in standard media (alpha-MEM (αMEM, Invitrogen) with 20% FBS, 1% P/S, 1% sodium pyruvate and 1% GlutaMAX (Invitrogen)). Cells were seeded in T75 flasks at a density of approximately 105 cells/cm^2^ and cultured in hypoxic conditions (5% O_2_, 5% CO_2_) to maintain their multipotency. Upon reaching 80% confluency, cells were passaged and split 1:4 in new flasks.

Cells were cultured in α-minimum essential medium (αMEM; Invitrogen) supplemented with 20% (*v*/*v*) defined fetal calf serum (Invitrogen), 1%, l-glutamine (Invitrogen), 1% sodium pyruvate (Invitrogen), 100 U/mL penicillin, and 100 μg/mL streptomycin (Invitrogen) as the standard growth media. Osteogenic growth media included 10 mM β-glycerophosphate, 0.1 mM ascorbate-2-phosphate, and 100 nM dexamethasone. Cells were maintained at 37 °C in a humidified 5% CO_2_ atmosphere. Cell culture media was changed every 3 days.

### 2.6. Cell Metabolic Activity—MTT Assay

MTT assay of BM-MSC treated with PLGA-MSV microspheres was performed to quantify metabolic activity [8]. Two thousand and five hundred BM-MSCs were seeded and cultured in a 24-cell culture well plate in the presence of the PLGA and PLGA-MSV microspheres (BM-MSCs:Particles, 1:5). Cells only were used as a control. MTT (3-(4,5-Dimethylthiazol-2-yl)-2,5-diphenyltetrazolium bromide) assay was performed on day 1, 4, and 7. Cell culture media was removed from cell culture wells and 500 µL of MTT working solution (0.5 mg/mL) were added into the wells. The cells were incubated in the MTT working solution at 37 °C for 4 h. The solution was removed from the cell culture wells and replaced with 500 µL of dimethyl sulfoxide (DMSO; Sigma Aldrich). The cells were incubated with DMSO at room temperature for 30 min. The solutions were transferred to a 96 well plate and the absorbance of the colored solutions was quantified by a spectrophotometer (Synergy H4 Hybrid Reader, BioTek, Winooski, VT, USA) at 570 nm. DMSO was used as blank. Cells only and PLGA particle wells were used as controls.

### 2.7. Alkaline Phosphatase (ALP) Activity Assay

Osteogenic differentiation was measured by ALP activity, a biomarker of osteoblastic differentiation. The assay was carried out according to manufacturer guidelines for the spectrophotometric procedure using ALP reagent (Vector Laboratories, Inc. Burlingame, CA, USA). The BM-MSCs were plated into a 24 well cell culture plate at a density of 2500 cells per well with the PLGA-MSV microspheres (20% *w*/*v*) loaded with BMP-2 was used as the experiential group. Cells alone, cells cultured with BMP-2, and empty PLGA-MSV microspheres without BMP-2 (20% *w*/*v*) were used as controls. Cell culture media was changed every 3 days. Cells were cultured in α-minimum essential medium (αMEM; Invitrogen) supplemented with 10% (*v*/*v*) fetal calf serum (Invitrogen), 100 U/mL penicillin, and 100 μg/mL streptomycin (Invitrogen) as the standard growth media. Culture conditions were 37 °C in a humidified 5% CO_2_ atmosphere. The osteogenic growth media included 10 mM β-glycerophosphate, 0.1 mM ascorbate-2-phosphate, and 100 nM dexamethasone. Cell culture media was replenished twice weekly. Cells were cultured in standard media until 60% confluence and then switched to osteogenic media. ALP assays were performed at week 1, 2, and 3. The medium was aspirated and 1 mL of PBS was added into each well to wash the cells. Cells were washed 3 times with PBS, and fixed in 10% buffered formalin for 15 min. Cells were then washed twice in deionized (DI) water and covered in ALP stain made fresh. ALP staining stock solution was made from ALP substrate kit III (Vector Laboratories. Inc., Burlingame, CA, USA) into 5 mL of 100 mM tris-HCl (pH = 8.2) solution. Absorbance of cells after staining was used to quantify ALP activity. The experiments were performed in triplicate.

### 2.8. Von Kossa Co-Staining

Cell cultures were co-stained for calcium-triphosphate mineral deposition by Von Kossa staining. Co-staining of the cell culture was performed to simultaneous both quantify ALP activity and provide a qualitative assessment of mineral deposition. Following ALP staining, cells were washed twice in DI water and soaked in in 1% aqueous silver nitrate (AgNO_3_) and placed under ultraviolet (UV) light for 60 min and then rinsed with DI water. To remove unreacted silver, 5% sodium thiosulfate was added for 5 min, removed and cells rinsed in DI water. Following this cell nuclei were counterstained with Nuclear Fast Red (Sigma Aldrich) for 5 min, rinsed in DI water, and serially dehydrated prior to characterization.

## 3. Results

### 3.1. PLGA-MSV Characterization

**Figure 1:**
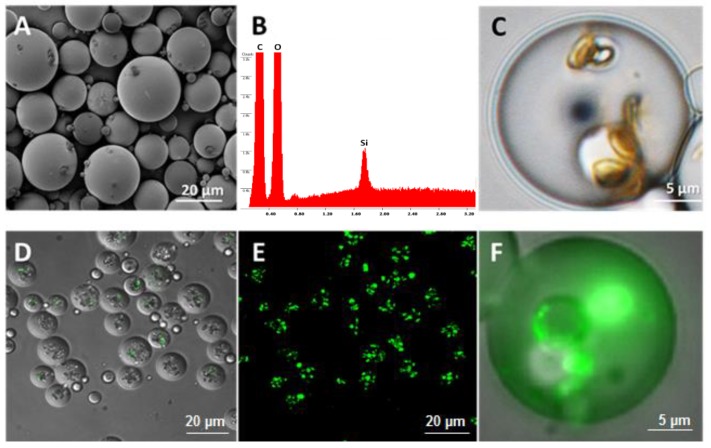
SEM and confocal images of PLGA-multistage vector (MSV). Representative SEM image of PLGA-MSV (**A**). EDX spectrum of PLGA-MSV microspheres showing the presence of the silicon peak corresponding to MSV (**B**). Confocal laser microscopy Z-stack of PLGA-MSVs loaded with FITC-BSA (in green), showing that MSVs were fully encapsulated into the PLGA microsphere (**D**,**E**). Close up of a PLGA-MSV (**C**,**F**).

The PLGA-MSVs were characterized by SEM-energy-dispersive X-ray (EDX) and confocal microscopy. SEM images show that the spherical-shaped PLGA-MSVs with smooth surfaces had a wide distribution from a few microns to approximately 50 μm, with an average diameter of 23 ± 3 (*n* = 50; Figure 1A). EDX spectrum showed the presence of a Si peak (Figure 1B), which indicates the presence of MSV particles inside of the PLGA microspheres. The full encapsulation of MSV was further confirmed by optical microscopy, where hemispherical MSVs appeared yellow in color (Figure 1C). MSVs were also loaded with a reporter protein (FITC-BSA) and imaged by confocal microscopy. Imaging clearly demonstrated that the FITC-BSA loaded MSVs (in green) were fully encapsulated (Figure 1D–F).

### 3.2. PLGA-MSV Loading with BMP-2 and In Vitro Release

**Figure 2:**
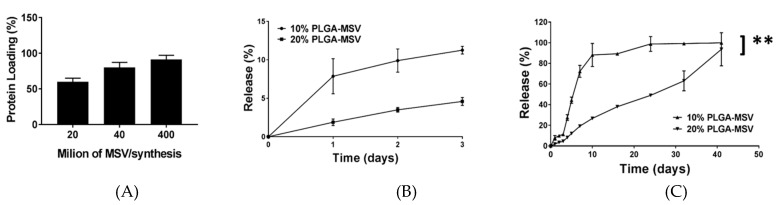
The in vitro loading and release profiles of BMP-2 using PLGA-MSV microspheres (10%, 20% *w*/*v*; *n* = 3). (**A**) The loading profiles of BMP-2 into MSV particles with fixed BMP-2 concentrations and mass, and varied MSV microparticle number. Cumulative release profile of BMP-2 from different 10% and 20% PLGA-MSV in the first three days (**B**) and over 41 days, (**C**). Values are reported as mean ± standard deviation. A value of *p* < 0.05 was considered statistically significant: ** *p* < 0.01 (calculated by *t* test analysis).

Recombinant human BMP-2 was used in this study. BMP-2 is a 29 kDa protein with an isoelectric point of 8.21 (Peprotech). The BMP-2 loaded PLGA-MSV microspheres (with a PLGA coating of 10% and 20% *w*/*v*) were used for in vitro sustained release studies. The loading efficiency of BMP-2 into MSV particles is shown in Figure 2A. Consistent with mass transport theory, the greater number of particles added to the solution of BMP-2, the greater amount of loading achieved and thus a higher loading efficiency. The in vitro release profiles of BMP-2 from different types of microspheres were monitored for 40 days. A delayed burst release over three days was demonstrated by 10% *w*/*v* PLGA-MSV microspheres (Figure 2B). When monitored to 40 days 90% of release was achieved within 10 days and sustained release equilibrium near 24 days (Figure 2C). In contrast, BMP-2 release from 20% *w*/*v* PLGA-MSV microspheres showed a more linear-like release with a more linear early release profile (Figure 2B). This slower rate of BMP-2 release continued until equilibrium was reached at 41 days (Figure 2C).

### 3.3. Cell Metabolic Activity—MTT Assay

**Figure 3:**
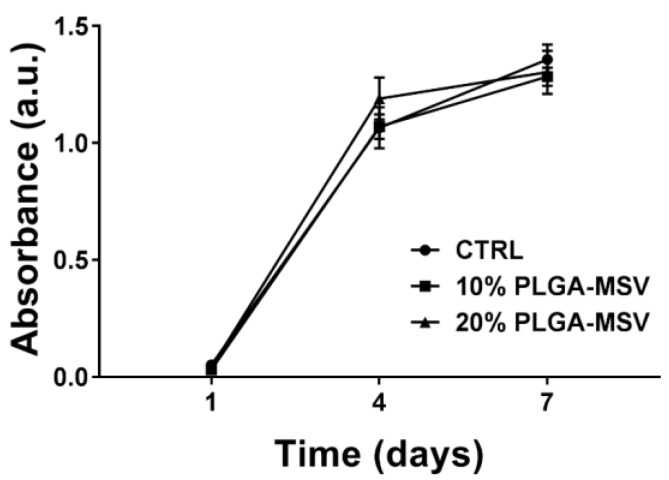
MTT assay for rat bone marrow stromal cells (BM-MSCs) cultured with 10% PLGA-MSV and 20% PLGA-MSV microspheres over 7 days (*n* = 3). Cell viability is reported as absorbance at 490 nm. The cell metabolic activity of BM-MSCs in the presence of both formulations of PLGA-MSV was comparable to that of untreated cells (CTRL). Values are reported as mean ± standard deviation. No statistical significance was found among selected experimental conditions.

Cell metabolic activity in the presence of the PLGA and PLGA-MSV microspheres was analyzed using MTT assay. Figure 3 shows the results of MTT assay for BM-MSCs cultured with PLGA-MSV microspheres at 1, 4, and 7 days. Metabolic activity among cells alone (control) and cells with the PLGA-MSV microspheres shared the similar trend and there was no significant difference among of the three groups at 1, 4, or 7 days. Cells in each group achieved a comparable metabolic activity and reached equilibrium at each time point.

### 3.4. PLGA-MSV-BMP2 Enhances Alkaline Phosphatase Activity (ALP)

**Figure 4:**
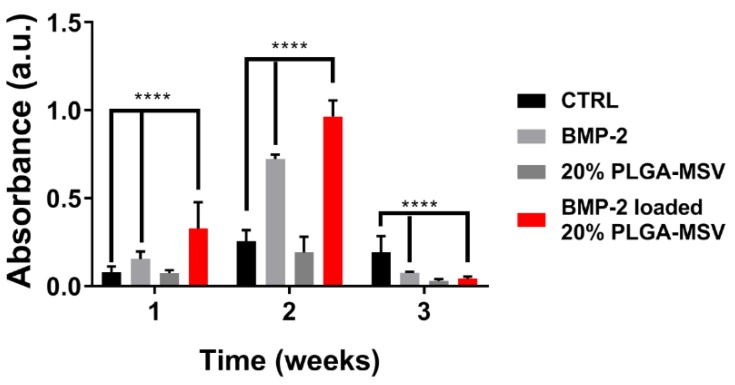
ALP activity of untreated BM-MSCs (CTRL), BM-MSCs treated with soluble BMP-2 or 20% PLGA-MSV loaded or non-loaded with BMP-2 over 3 weeks (mean ± SD, *n* = 3). Values are reported as mean ± standard deviation. Data was analyzed through 2-way ANOVA. A value of * *p* < 0.05 was considered statistically significant (**** *p* < 0.0001).

ALP is synthesized by osteoblasts and is a biomarker of presumed ECM production during BM-MSC differentiation [24]. As shown in Figure 4, at weeks 1 and 2, BMP-2 loaded 20% PLGA-MSV(PLGA-MSV-BMP2) microspheres showed higher ALP activities compared to cells alone and cells treated with BMP-2 (BMP2). At week 2, cells treated with PLGA-MSV-BMP2 showed the highest ALP activity during the duration of the investigation. The ALP activities decreased to a fairly low level for all controls and experimental groups after 3 weeks. Although, interestingly, the ALP activity of the control remained elevated at this time point.

### 3.5. ALP Staining and Von Kossa Staining

**Figure 5:**
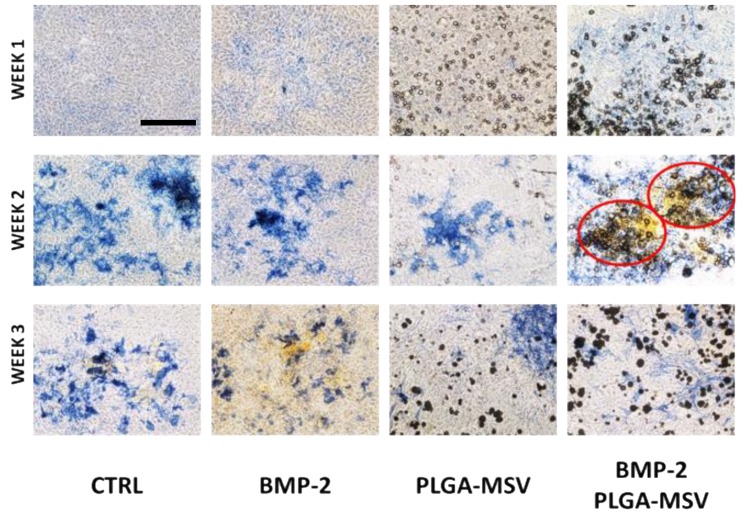
ALP and Von Kossa co-staining of BM-MSCs and BM-MSCs with experimental groups. ALP activities increased from week 1 to week 2 and then decreased from week 2 to week 3 in BMP2 and BMP2-PLGA-MSV. The cells in the presence of the PLGA-MSV-BMP-2 microspheres at week 2 showed the strongest ALP activity (red circles) among all of the control and experimental groups over the 3-week culture period. Von Kossa staining progressively increased in all groups with BMP2 and BMP2-PLGA-MSV showing the most staining at week 3 (Scale bar: 60 μm).

All groups were co-stained for ALP and calcium using standard ALP staining and Von Kossa stain. ALP staining data of BM-MSC treated with BMP2 and PLGA-MSV-BMP2 correlated with ALP quantitative activity data. BMP2 and PLGA-MSV-BMP2 showed qualitatively increased staining at 1 and 2 weeks while untreated cells showed increased ALP staining at 3 weeks (Figure 5).

Von Kossa staining was performed to assess functional activity of BM-MSC osteoinduction. Calcium deposition was delayed compared to ALP staining. Calcium (black stain) is seen in the PLGA-MSV-BMP2 group as early as one week and continues to increase throughout the 3 weeks having the most deposition of all groups. In the control and BMP2 group calcium begins to be seen at 2 weeks yet the deposition of calcium only continues to increase in the BMP2 group. PLGA-MSV did not demonstrate appreciable deposition until week 3 (Figure 5).

## 4. Discussion

This study reports the development and investigation of a PLGA based protein delivery system, PLGA-MSV microspheres, for controlled protein release to stimulate cell differentiation. Herein, we studied the loading and release of BMP-2 from MSV, the relationship of PLGA:BMP concentration on BMP-2 release, and the effect of long-term constant BMP-2 stimulation on BM-MSCs. BMP-2 was successfully encapsulated into the microsphere system and the performance was tested in vitro. The experiments show that: (1) BMP/PLGA-MSV microspheres had the ability to release small but effective doses of BMP-2 for 40 days in a controlled, linear fashion; (2) the release profile of the BMP/PLGA-MSV microspheres was dependent on the PLGA coating of 10% or 20%; (3) the PLGA-MSV system did not impact cell metabolic activity; and (4) BMP-2 released from PLGA-MSV microspheres was capable of osteoinduction of BM-MSCs.

Previously, published reports with the clinical and pre-clinical use of BMP-2 have demonstrated bone augmentation, but the growth occurred in an uncontrolled and non-physiologic fashion. Partly due to the short half-life and need for supraphysiologic dosing, this resulted in heterotopic bone formation. Clinically this has translated in to the development of multiple complications [1,10]. Currently formulated BMP devices must be used at high concentrations resulting in problems with protein release timing stability, and need for accessory factors [11,12]. Additionally, the bone regenerative process under these conditions is very different from the normal physiologic processes in normal bone healing in which multiple BMPs and other growth factors are present in overlapping temporal patterns [13,14]. Here we reported a biomolecular release vehicle capable of controlling release kinetics of an encapsulated molecule for localized protein delivery, in this case BMP2. This phenomenon is affected by the percentage of PLGA used indicating an added level of spatiotemporal control of the system. Increasing PLGA percentage slowed release kinetics from a burst release to a more constant release and could theoretically lead to more prolonged and slowed release kinetics [25,26]. The slower release kinetics may be more biologically accurate and induce a more sustained response in contrast to the massive burst release kinetics often seen from PLGA alone [27]. Here we demonstrated this system is capable of long-term delivery as well as in vitro osteoinduction.

As many of the issues attributed to the use of growth factors in regenerative medicine have been centered on the lack of controlled release, this model presents a system for localized delivery within a clinically relevant scenario such as a healing fracture. MSCs are the major regenerative cell involved in the fracture healing process [28]. Their stimulation is paramount for the successful regeneration of tissue and organized bone formation. Without their coordinated efforts through properly induced pathways, dysregulated and disorganized growth occurs leading to weakened fracture callus formation and recurrent injuries [15,16]. Previously, investigators have demonstrated the efficacy of BMP2 release on the activity of MSCs [17]. Here we show that normal cellular function is not inhibited by the PLGA-MSV system and that sustained BMP-2 release results in MSC osteoinduction and increased ALP activity compared to BMP-2 alone. Cells can proliferate normally in the presence of the PLGA-MSV microspheres, which, indicates that these materials were nontoxic to cells and compatible with normal cell function. When loaded with BMP, the PLGA-MSV system was used to stimulate BM-MSC in vitro. ALP enzyme activity was increased in the PLGA-MSV-BMP-2 group and this correlated with ALP staining. These findings translated to a persistent and increasing deposition of calcium seen in the Von Kossa co-stain. The delayed deposition of calcium is consistent with normal biological function and an expected outcome [29]. The PLGA-MSV-BMP system induced early and robust ALP activity at one week compared to BMP2 alone. At this same time point calcium is already detected in the Von Kossa stain of the PLGA-MSV-BMP2 group. This suggests that the slowed release may in fact have greater biological activity compared to high dose free BMP. This localized effect may provide insights into the further concomitant use of MSCs and PLGA-MSV vectors for regenerative purposes in other applications.

There are several limitations with the current study. First the lack of free PLGA-BMP2 makes direct comparison difficult. There have been several studies looking at the use of PLGA as a protein carrier and more specifically a BMP2 carrier. These studies reported the release kinetics of BMP2 from PLGA and its impact in vitro on osteoinduction. Given the historical findings with PLGA alone as the carrier we chose to focus on the use of MSV encapsulated in PLGA. Second, while ALP activity, staining and calcium deposition all form part of the evaluation of BM-MSC osteoinduction further studies in the transcriptional changes and more quantitative histological assessments could be performed. This was out of the scope of this initial work and is the focus of subsequent works in progress. Third, an additional comparison group looking into the effects of PLGA-MSV ratio on bioactivity toward BM-MSC is needed. While there were significant differences between the release profiles between 10% and 20% PLGA-MSV-BMP2 no biologic data was obtained. This is currently a subject of current investigation. Lastly, this is only an in vitro study with one cell line. The biologic milieu of the in vivo micro-environment is composed of a complex interplay of signaling and cell types. While our in vitro findings are promising they warrant analysis in an in vivo fracture model to assess the efficacy of localized controlled release.

## 5. Conclusions

In conclusion, this biocompatible, biodegradable, and osteogenic PLGA-MSV microsphere system holds promise as a candidate for the delivery of safe, local, small but effective doses of bioactive proteins for pharmaceutical induction of osteoregeneration. The local and long-term delivery of proteins and other bioactive molecules for orthopedic applications may avoid the complications associated with burst release and supraphysiologic dosing of BMP-2 currently afforded by available delivery systems.
